# SynergyFit energy recovery framework for human-centric power generation in multi-modal fitness environments

**DOI:** 10.1038/s41598-025-21217-w

**Published:** 2025-10-27

**Authors:** Douglas Yeboah, Bismark Danso Annor

**Affiliations:** Department of Renewable Energy Engineering, University of Mines and Technology, Tarkwa, Ghana

**Keywords:** Multi-modal exercise systems, Exercise energy harvesting, Theoretical modeling, Smart gym infrastructure, Renewable energy integration, Physiology, Climate sciences, Cardiology, Energy science and technology, Engineering, Physics

## Abstract

The demand for sustainable energy solutions has driven interest in capturing power from physical activity in fitness settings. This study introduces the SynergyFit Energy Recovery Model, a comprehensive theoretical framework for estimating and optimizing energy recovery across resistance, cardiovascular, and functional training. The model addresses the challenge of effectively harvesting energy in multi-modal exercise environments by identifying distinct recovery patterns: resistance training provides steady yields through consistent force application, while cardiovascular and functional training scale more rapidly with duration due to dynamic movement profiles. Validation against reported energy recovery values confirms the model’s strong predictive robustness and alignment with established trends. For a training session with an average mass (plate and trainer) of 80 kg, the energy recovery results are as follows: Resistance training yields energy recoveries from 9324 J at 1.5 min to 4827.76 J at 5.0 min; Cardio training shows a marked increase, with energy recoveries ranging from 860.82 J at 1.5 min to 31,882.50 J at 5.0 min; Functional training produces energy recoveries from 466.93 J at 1.5 min to 7130.64 J at 5.0 min. The total energy recovery across these modalities ranges from 10,651.76 J to 43,840.90 J, depending on the exercise duration. The sensitivity analysis further demonstrates that increasing exercise mass, higher cardio training velocities, and longer resting periods significantly enhance energy recovery. Notably, cardio training exhibits the highest energy recoverable potential, with cumulative recovery reaching approximately 43.8 kJ per session. These insights highlight the critical role of optimizing exercise duration and parameters to maximize energy recovery. Overall, the novel SynergyFit Energy Recovery Model offers a significant advancement in understanding and improving energy recovery in fitness environments. By integrating multiple exercise modes, this model contributes to more efficient energy utilization and underscores the benefits of parameter optimization for enhanced sustainability in fitness facilities.

## Introduction

 Human-centered energy harvesting is increasingly recognized as a complementary pathway in the global transition toward renewable energy, particularly through small-scale, distributed power generation^1^. Within this context, the fitness sector represents a promising frontier, where structured and repetitive human motion can be transformed into usable electricity^2^. Unlike industrial waste heat recovery or vibration harvesting in transport and construction^3,4^, energy recovery in gyms directly integrates human activity with renewable energy production, aligning sustainability goals with user engagement^5,6^. Globally, the fitness industry serves over 200 million people across more than 210,000 facilities, representing a vast, yet underutilized, opportunity for distributed energy harvesting^7^. Even modest energy capture from daily exercise routines could collectively offset significant portions of gym operational demand.

Beyond fitness centers, the broader sports sector is also emerging as a key domain for energy recovery innovation. For instance, Yeboah et al.^8^ demonstrated a hybrid energy system at the Tarkwa Akoon Soccer Park in Ghana, where solar photovoltaic and piezoelectric technologies were combined to simultaneously capture energy from solar radiation and the dynamic movements of players. Similarly, Zhou et al.^9^ explored biomechanical systems that utilize heel strikes, limb swings, and surface vibrations to generate real-time electricity. These examples underscore the potential of sports and exercise environments as scalable platforms for sustainable energy recovery. Importantly, such implementations not only provide renewable energy but also promote public awareness, behavioural change, and community engagement in sustainability initiatives.

Modern fitness facilities are particularly well suited for such innovations, as they incorporate resistance training stations, cardiovascular machines, and functional training systems^10^. The structured and repetitive nature of exercise provides consistent mechanical inputs that can be converted into electricity^11^. Multi-modal exercise environments—where users engage in strength, endurance, and mobility training—further enhance this potential by offering varied movement patterns and stable energy flows^12^. From a systems perspective, this diversity mitigates intermittency issues common in other renewable sources, such as solar and wind, by ensuring more continuous energy generation during peak gym usage hours. This convergence of physical engagement and technological integration positions fitness environments as scalable, untapped sites for sustainable energy generation. Beyond offsetting operational energy demands, these systems contribute to broader environmental goals by fostering circular energy flows and reducing the carbon footprint of recreational spaces.

Several technologies already demonstrate the feasibility of exercise-based energy harvesting. Cardio machines such as treadmills, elliptical trainers, and stationary bikes can be equipped with mechanisms that convert user motion into electricity, which can then be stored or used to power auxiliary systems within the facility^13^. Resistance machines, including weightlifting stations and cable pulley systems, similarly allow energy recovery from controlled weight movements^14,15^. Functional training devices such as rowing machines and cross-trainers harness natural biomechanics to generate usable power^14^. A practical example is provided by The Great Outdoor Gym Company, which developed an installation in Hull, United Kingdom, that has generated more than 40,000 W-hours of electricity while simultaneously promoting public health and community engagement^16^. Commercially available products, such as SportsArt’s Eco-Powr™ cardio equipment, further underscore the maturity of the field, with reported energy conversion efficiencies of 74–80%, highlighting the readiness of such systems for broader adoption^5^.

Beyond machine-based harvesting, ambient vibrations within gym environments present additional opportunities for piezoelectric and electromagnetic conversion^17^. Saeed et al.^18^ proposed retrofitting conventional exercise equipment with these technologies to reduce reliance on external power sources. Stefanyshyn and Wannop^19^ emphasized the importance of athlete–equipment interactions in enhancing recovery efficiency, while Artioli et al.^20^ examined energy system contributions during exercise, providing insights into aligning biomechanical output with harvesting potential. Other studies have explored innovative prototypes, including energy-generating bicycles^21–24^, flywheel-generator systems in pull-down machines^25^, and superconducting technologies for rotating fitness equipment^26^. Collectively, these studies reflect a growing body of work advancing exercise-driven energy recovery. When combined with recent advances in energy storage technologies, such as high-capacity supercapacitors and solid-state batteries, these systems open pathways for capturing and storing intermittent exercise-generated energy, enabling fitness centers to integrate harvested electricity into building-level microgrids.

Despite these promising developments, most prior studies have concentrated on individual machines, leaving a critical gap in understanding integrated energy recovery across multi-modal exercise environments. Existing research largely isolates resistance, cardiovascular, or functional equipment, overlooking how these modalities can interact synergistically to maximize recovery efficiency^15,18^. In contrast, multi-modal exercise settings offer ideal conditions for collective harvesting, where diverse movement patterns and consistent mechanical inputs generate higher and more stable outputs than single devices alone. This transition from isolated device-level analysis to a holistic systems perspective is essential for scaling energy-harvesting gyms into meaningful contributors to sustainable urban energy ecosystems.

The novelty of this research lies in the development of the SynergyFit Energy Recovery Model, a unified theoretical framework designed to evaluate resistance, cardiovascular, and functional training simultaneously within multi-modal fitness environments. Unlike previous studies centered on isolated prototypes, the SynergyFit model explicitly captures cross-modal interactions, quantifies their combined energy potential, and applies sensitivity analysis to optimize key training parameters. This integration across modalities, combined with a generalized, scalable model validated against literature data, constitutes a significant advance over previous studies and directly addresses the research gap in holistic gym energy recovery systems.

By moving beyond single-machine prototypes and reframing energy harvesting in terms of synergy across multiple exercise modalities, this study provides new insights for designing energy-efficient fitness facilities. The findings aim to guide the development of next-generation gyms as micro-scale renewable energy hubs that simultaneously advance sustainability, reduce operational costs, and promote active lifestyles. More broadly, this work positions fitness-based energy recovery as a novel contributor within the distributed renewable energy landscape, with implications for sustainable urban design, decentralized power generation, and the integration of human activity into future energy systems.

## Theoretical framework for human energy recovery in multi-modal training

This section presents the SynergyFit Energy Recovery Model, a unified theoretical framework designed to quantify recoverable electrical energy across resistance, cardiovascular, and functional training modalities. Rooted in principles of classical mechanics and biomechanics, the model derives energy output using modality-specific force–displacement and power–time relationships, thereby capturing the unique dynamics of each exercise form.

The framework integrates key physiological and mechanical parameters, including mass, velocity, displacement, and rest intervals, while introducing synergistic efficiency terms to account for real-world conversion losses and system non-idealities. These components are systematically coupled into a scalable formulation that estimates total energy recovery potential within multi-modal training environments.

To establish robustness, the section proceeds by deriving sub-models for individual modalities, formulating hypotheses that govern their combined performance, and benchmarking the integrated framework through mathematical simulations under standardized gym conditions. Sensitivity analyses are then conducted to identify the most influential performance variables, providing insight into how design and training parameters can be optimized to maximize energy harvesting efficiency.

### Energy recovery model for resistance training

To determine the energy recovery potential of resistance training and equipment, the work done, $\:W$, during the training session is calculated by integrating the force function $\:F\left(x\right)$ over distance (displacement) $\:x$, from the following governing equation^27^:

1 $$\:W=F\times\:d$$

where, $\:W\:$ is the work done, $\:\:F\:$is the force applied, and $\:d$ is the distance covered by the applied force.

Given a set of discrete data points or a complicated function of force $\:F\left(x\right),\:$the Analytical Integration method is used^28^.

For $\:n$ nodes and weights $\:\left({x}_{i},\:{w}_{i}\right)$:

2 $$\:\:W\approx\:{\sum\:}_{i=1}^{n}{w}_{i}\:F\left({x}_{i}\right)$$

Since integrating force over displacement gives work done, Eq. (2) can be rewritten as;

3 $$\:W={\int\:}_{{x}_{1}}^{{x}_{2}}F\left(x\right)dx$$

Assumptions:

1. $\:F\left(x\right)$is a first-order polynomial function such that $\:F\left(x\right)={a}_{0}+{a}_{1}x$;
2. Consider the number of reps of training as well as resting time, training momentum, and force exerted.

$$\:W={\int\:}_{{x}_{1}}^{{x}_{2}}\left({a}_{0}+{a}_{1}x\right)dx\:$$

4 $$\:W={\left[{a}_{0}x+{a}_{1}\frac{{x}^{2}}{2}\right]}_{{x}_{1}}^{{x}_{2}}$$

Employing the Analytical Integration method, Eq. (4) is simplified as:

5 $$\:W=\left({a}_{0}\left({x}_{2}-{x}_{1}\right)+{a}_{1}\frac{{(x}_{2}^{2}-{x}_{1}^{2})}{2}\right)$$

where, $\:{a}_{0}\:$ is the momentum function of displacement for resistance training, $\:{a}_{1}\:$ is the force function of displacement for resistance training, $\:{x}_{1}\:$ is the initial displacement, and $\:{x}_{2}\:$ is the initial displacement.

The momentum function of resistance training represents the intensity of exercise. Exercise intensity is given as:

6 $$\:{a}_{0}=\:{m}_{0}v.\frac{N}{{R}_{T}}$$

where, $\:{a}_{0}\:$ is the momentum function of displacement for resistance training, $\:{m}_{0}\:$ is the mass of plates to be lifted, $\:v\:$ is the velocity of weight lifting, $\:N\:$ is the number of reps for each training round, and $\:{R}_{T}\:$ is the initial displacement.

The force function of displacement for resistance training represents the resistance level considering technical parameters such as mass of plates, velocity, duration of exercise, and differential displacement constant ($\:\sigma\:$) which accounts for the portion of the total displacement occupied by the plate stack.

7 $$\:{a}_{1}=\:{m}_{0}.\frac{dV}{dT}.\frac{1}{\sigma\:}$$

where, $\:{a}_{1}\:$ is the force function of displacement for resistance training, $\:{m}_{0}\:$ is the mass of plates to be lifted, $\:dV\:$ is the velocity of weight lifting, $\:dT\:$ is the training duration, and $\:\sigma\:\:$ is the initial displacement.

Combining Eqs. (5), (6), and (7), the resistance training model becomes:

8 $$\:W=\:\left({m}_{0}v.\frac{N}{{R}_{T}}\text{}\left({x}_{2}-{x}_{1}\right)+\:{m}_{0}.\frac{dV}{dT}.\frac{{(x}_{2}^{2}-{x}_{1}^{2})}{2\sigma\:}\right)$$

According to the law of conservation of energy, mechanical work can be converted into electrical energy^29,30^ as shown in Eq. (9):

9 $$\:{E}_{\text{E}\text{l}\text{e}\text{c}}=\eta\:\times\:W$$

where, $\:{E}_{\text{E}\text{l}\text{e}\text{c}}$ is the electrical energy, $\:\eta\:$ is the efficiency of the generator, and $\:W$ is the mechanical work done.

From Eq. (9), and considering the synergistic efficiency ($\:{\eta\:}_{sys.}^{R}$), of resistance training, Eq. (8) becomes:

10 $$\:{E}_{R}=\:{\eta\:}_{sys.}^{R}\left({m}_{0}v.\frac{N}{{R}_{T}}\text{}\left({x}_{2}-{x}_{1}\right)+\:{m}_{0}.\frac{dV}{dT}.\frac{{(x}_{2}^{2}-{x}_{1}^{2})}{2\sigma\:}\right)$$

where, $\:{E}_{R}$ is the Energy produced from resistance training, $\:{\eta\:}_{sys.}^{R}$ is the synergistic efficiency of resistance training, $\:{m}_{0}\:$ is the mass of plates to be lifted, $\:v\:$ is the velocity of weight lifting, $\:N\:$ is the number of reps for each training round, $\:{R}_{T}\:$ is the resting time, $\:dV\:$ is the velocity of weight lifting, $\:dT\:$ is the training duration, and $\:\sigma\:\:$ is the initial displacement.

The resistance training model is based on the work done during the lifting process, represented by the force-displacement relationship. The derived model considers factors such as the number of repetitions, resting time, and training momentum. The resulting expression (Eq. 10) quantifies the electrical energy recoverable from resistance training, incorporating the synergistic efficiency of the system.

### Energy recovery model for cardio training

To determine the energy recovery potential of cardio training and the use of cardio training machines, power output $\:P\left(t\right)$ is integrated over time $\:t$, from the following governing equations^31^:

11 $$\:P=\frac{W}{t}$$

where, $\:P$ is the power output, $\:W$ is the work done, and $\:t$ is the time taken.

This equation can be rewritten in terms of force $\:\left(F\right)$, distance $\:\left(d\right)$ and time $\:\left(t\right)$ since the product of force and distance (displacement) is work done.

$$\:P=F.\frac{d}{t}$$

The expression $\:\frac{d}{t}$ also represents displacement as a ratio to time which is velocity. Hence the governing Equation can be further expressed as the product of the force function of time and velocity function of time becomes Eq. (12)^32,33^:

12 $$\:P\left(t\right)=F\left(t\right)\times\:v\left(t\right)$$

where, $\:P\left(t\right)$ is the instantaneous power at time $\:t,$ $\:F\left(t\right)$ is a force function at time $\:t$, and $\:v\left(t\right)$ is a velocity function of time$\:\:t.$.

If $\:F\left(t\right)$ and $\:v\left(t\right)$ are simple functions such that $\:F\left(t\right)=at\:$and $\:v\left(t\right)=bt,$ then the Analytical Integration method is employed for the following function.

13 $$\:P\left(t\right)=at\times\:bt=ab{t}^{2}$$

$$\:E={\int\:}_{0}^{T}P\left(t\right)dt$$

$$\:E={\int\:}_{0}^{T}ab{t}^{2}dt$$

$$\:E{=\left[\frac{ab}{3}{t}^{3}\right]}_{0}^{T}$$

14 $$\:\:\:E=\:\frac{ab}{3}{T}^{3}$$

To avoid overestimating energy output, resting intervals between cardio routines are incorporated into the model. Given that energy scales cubically with time, the square of the resting interval is applied as a correction factor to normalize the effective training duration^34^.

Equation (14) becomes;

15 $$\:E=\frac{ab}{3{{R}_{T}}^{2}}{T}^{3}$$

Applying the concept of energy conversion from Eq. (9) with the synergistic efficiency ($\:{\eta\:}_{sys.}^{C}$) for cardio training, Eq. (15) becomes:

16 $$\:{E}_{C}={\eta\:}_{sys.}^{c}\frac{ab}{3{{R}_{T}}^{2}}{T}^{3}$$

where, $\:{E}_{C}$ is the energy produced from cardio training, $\:{\eta\:}_{sys.}^{C}$ is the synergistic efficiency of cardio training, $\:a\:$ is the force function of cardio training, $\:b\:$ is the velocity function of cardio training, $\:{R}_{T}\:$ is the resting time, and $\:T\:$ is the training duration.

The cardio training model derives energy recovery based on power output, which is a function of force and velocity over time. The final expression (Eq. 16) integrates the training duration and resting intervals to estimate the energy produced during cardio sessions, adjusted by the system’s synergistic efficiency.

### Energy recovery model for functional training

Functional training modes are characterized by the interplay of both potential and kinetic energy due to dynamic movements and load-bearing postures during training sessions and equipment usage^35^. These exercises typically involve repeated lifting, swinging, or bodyweight-driven actions, each contributing to changes in speed and elevation, thereby activating kinetic and potential energy systems. To estimate the energy recovery potential in functional training environments, the total mechanical energy, comprising both potential and kinetic components, is calculated and integrated over the duration of the exercise session^36^.

The governing equations for these energy forms are as follows:

Kinetic energy equation^27^:

17 $$\:{E}_{\text{k}\text{i}\text{n}\text{e}\text{t}\text{i}\text{c}}\left(t\right)=\frac{1}{2}{mv\left(t\right)}^{2}$$

where, $\:{E}_{\text{k}\text{i}\text{n}\text{e}\text{t}\text{i}\text{c}}\left(t\right)$ is the kinetic energy, $\:m$ is the mass, and $\:v$ is the velocity.

Potential Energy Eq. (31):

18 $$\:{E}_{\text{p}\text{o}\text{t}\text{e}\text{n}\text{t}\text{i}\text{a}\text{l}}\left(t\right)=mgh\left(t\right)$$

where, $\:{E}_{\text{p}\text{o}\text{t}\text{e}\text{n}\text{t}\text{i}\text{a}\text{l}}\left(t\right)$ is the potential energy, $\:m$ is the mass, $\:g$ is the gravity, and $\:h$ is the height.

If $\:v\left(t\right)$ and $\:h\left(t\right)$ are assumed to be linear time-dependent functions such that $\:v\left(t\right)=at\:$and $\:h\left(t\right)=bt,\:$ the Analytical Integration method is employed to determine the total recoverable energy over the training duration.

Equation (17) becomes:

19 $$\:{E}_{\text{k}\text{i}\text{n}\text{e}\text{t}\text{i}\text{c}}\left(t\right)=\frac{1}{2}{m{a}^{2}t}^{2}$$

Equation (18) also becomes:

20 $$\:{E}_{\text{p}\text{o}\text{t}\text{e}\text{n}\text{t}\text{i}\text{a}\text{l}}\left(t\right)=mgbt$$

Integrating the sum of potential and kinetic energy gives;

$$\:E={\int\:}_{0}^{T}\left({E}_{\text{k}\text{i}\text{n}\text{e}\text{t}\text{i}\text{c}}\left(t\right)+{E}_{\text{p}\text{o}\text{t}\text{e}\text{n}\text{t}\text{i}\text{a}\text{l}}\left(t\right)\right)dt$$

$$\:E={\int\:}_{0}^{T}\left(\frac{1}{2}{m{a}^{2}t}^{2}+mgbt\right)dt$$

$$\:E={\left[\frac{1}{2}m{a}^{2}\frac{{t}^{3}}{3}+mgb\frac{{t}^{2}}{2}\right]}_{0}^{T}$$

21 $$\:E=\frac{1}{6}m{a}^{2}{T}^{3}+\frac{1}{2}mgb{T}^{2}$$

For functional training, which involves combined kinetic and potential energy contributions, the model accounts for trainer rest periods to ensure realistic energy estimations. As energy depends on a cubic function of time, the cube of the resting time is introduced as a divisor to adjust for non-continuous activity durations^34^.

Equation (21) becomes:

22 $$\:E=\frac{1}{6{{R}_{T}}^{3}}m{a}^{2}{T}^{3}+\frac{1}{2{{R}_{T}}^{2}}mgb{T}^{2}$$

Applying the concept of energy conversion in Eq. (9) with the synergistic efficiency ($\:{\eta\:}_{sys.}^{F}$) for functional training leads to the following expression for the total energy produced during functional training sessions:

23 $$\:{E}_{F}=\:{\eta\:}_{sys.}^{F}\left(\frac{1}{6{{R}_{T}}^{3}}m{a}^{2}{T}^{3}+\frac{1}{2{{R}_{T}}^{2}}mgb{T}^{2}\right)$$

where, $\:{E}_{F}$ is the energy produced from functional training, $\:{\eta\:}_{sys.}^{F}$ is the synergistic efficiency of functional training, $\:m\:$ is the mass of plates of functional machines, $\:a\:$ is the velocity of weight lifting, $\:g\:$ is the gravity, $\:b\:$ is the height through which weight is lifted, $\:{R}_{T}\:$ is the resting time, and $\:T\:$ is the training duration.

The functional training model considers both kinetic and potential energy contributions during exercises involving complex movements. The derived equation (Eq. 23) accounts for the mass, velocity, and height of the exercise, providing an estimate of the recoverable energy, again adjusted by synergistic efficiency.

### Unified multi-modal energy recovery model

Combining the contributions from the from resistance, cardio, and functional training modes, gives the total recoverable energy during a complete training session.

#### Total energy recovery model

24 $$\:{E}_{\text{R}\text{E}\text{C}}={E}_{R}+{E}_{C}+{E}_{F}$$

25 $$\:{E}_{\text{R}\text{E}\text{C}}={\eta\:}_{sys.}^{R}\left({m}_{0}v.\frac{N}{{R}_{T}}\text{}\left({x}_{2}-{x}_{1}\right)+\:{m}_{0}.\frac{dV}{dT}.\frac{{(x}_{2}^{2}-{x}_{1}^{2})}{2\sigma\:}\right)+{\eta\:}_{sys.}^{c}\frac{ab}{3{{R}_{T}}^{2}}{T}^{3}+{\eta\:}_{sys.}^{F}\left(\frac{1}{6{{R}_{T}}^{3}}m{a}^{2}{T}^{3}+\frac{1}{2{{R}_{T}}^{2}}mgb{T}^{2}\right)$$

By summing the contributions from resistance, cardio, and functional training models, the total energy recoverable during a complete training session is determined. The integrated model dubbed “SynergyFit Energy Recovery Model” (Eq. 25) offers a comprehensive approach to estimating overall energy recovery, crucial for optimizing energy harvesting in multi-modal training environments.

### Hypotheses derived from the synergyfit framework

The developed models form the base framework for calculations and mathematical simulations. The following hypotheses are formulated from the various models developed in this chapter.

#### Hypothesis 1: energy production in resistance training

The energy $\:{E}_{\text{r}\text{e}\text{s}\text{i}\text{s}\text{t}\text{a}\text{n}\text{c}\text{e}}$ produced during resistance training is directly proportional to the mechanical work done.

26 $$\:{H}_{1}:{E}_{\text{r}\text{e}\text{s}\text{i}\text{s}\text{t}\text{a}\text{n}\text{c}\text{e}}\propto\:{\int\:}_{{x}_{1}}^{{x}_{2}}F\left(x\right)dx$$

#### Hypothesis 2: energy production in cardio training

The energy $\:{E}_{\text{c}\text{a}\text{r}\text{d}\text{i}\text{o}}$ produced during cardio training increases as mechanical power output and exercise duration increase.

27 $$\:{H}_{2}:{E}_{\text{c}\text{a}\text{r}\text{d}\text{i}\text{o}}\propto\:{\int\:}_{0}^{T}P\left(t\right)dt$$

#### Hypothesis 3: energy production in functional training

The energy $\:{E}_{\text{f}\text{u}\text{n}\text{c}\text{t}\text{i}\text{o}\text{n}\text{a}\text{l}}$ produced during functional training is a function of the kinetic and potential energy involved in the dynamic movements during the training.

28 $$\:{H}_{3}:{E}_{\text{f}\text{u}\text{n}\text{c}\text{t}\text{i}\text{o}\text{n}\text{a}\text{l}}\propto\:\left(\frac{1}{2}{mv\left(t\right)}^{2}+mgh\left(t\right)\right)dt$$

#### Hypothesis 4: integrated multi-modal energy recovery

The total energy $\:{E}_{\text{R}\text{E}\text{C}}$ from an integrated multi-modal exercise environment is a cumulative function of the energy produced from each training modality.

29 $$\:{H}_{4}:\:{E}_{\text{R}\text{E}\text{C}}\propto\:\:{E}_{\text{t}\text{o}\text{t}\text{a}\text{l}}=\:{\eta\:}_{sys.}^{r}{\int\:}_{{x}_{1}}^{{x}_{2}}F\left(x\right)dx+{\eta\:}_{sys.}^{c}.{\int\:}_{0}^{T}P\left(t\right)dt+\:{\eta\:}_{sys\:}^{f}.{\int\:}_{0}^{T}\left(\frac{1}{2}{mv\left(t\right)}^{2}+{mgh\left(t\right)}^{2}\right)dt$$

These hypotheses are designed to be testable through computational simulations and, ultimately, through empirical validation in controlled training environments.

### Model validation via mathematical simulation

This section presents the validation of the proposed theoretical framework through structured mathematical simulations. The goal was to estimate the energy recoverable from resistance, cardio, and functional training modalities under realistic operating conditions. Simulations accounted for biomechanical input variables and equipment-specific factors, enabling the evaluation of each modality’s energy contribution and the system’s overall performance. This approach provided quantitative insights into the model’s applicability, robustness, and relevance for real-world energy recovery in multi-modal training environments.

#### Key assumptions and conditions for model simulations

The following assumptions were established to facilitate consistent and representative simulation outcomes:

- Concurrent training modalities: It is assumed that all three categories of training equipment—resistance, cardio, and functional—are used simultaneously, replicating a real-world gym environment involving multiple users.
- Standardized training load: Based on the biomechanical standards reported by^37^, an average trainer weight of 80 kg is assumed across all modalities. For resistance training, this mass is translated into external loads corresponding to machine plate stacks (10–100 kg), representing exercises such as chest press, lat pulldown, and leg extension. For functional training, the mass reflects bodyweight or loaded movements (20–100 kg), including rowing, push-pull actions, and cable-based exercises. For cardio training, the 80 kg body mass simulates a typical adult using treadmill or cycling equipment, ensuring alignment with manufacturer specifications for commercial machines.
- Repetition and resting protocols: Each training session is quantified by the number of repetitions per set and inter-set rest durations, following physiological norms established in the exercise science literature^38,39^. For resistance and functional training, 3 sets of ~ 10 repetitions are assumed, each lasting 30–60 s, with rest intervals ranging from 30 to 150 s depending on training intensity. For cardio training, protocols simulate steady-state running or cycling at velocities between 2.5 and 4.0 m/s for durations of 1.5–5.0 min, representing common treadmill and cycling workouts.
- Energy conversion assumption: The mechanical work performed during exercise is assumed to be transformed into electrical energy using high-efficiency generators, aligned with prior hybrid system studies^40^.

#### Key technical considerations for model simulations

Several engineering and human-centered parameters were considered during simulation:

- Resistance load: Defined as the external force applied or overcome during lifting or movement in resistance training.
- Training intensity and duration: Includes metrics such as speed, duration, number of repetitions, and compliance with safety and ergonomic standards^37^. In practical terms, this reflects standard gym prescriptions: moderate resistance (60–80% of one-repetition maximum) for resistance sets, steady-state cardio intensities corresponding to moderate exertion levels (50–70% VO₂ max), and functional training at variable intensities using compound, whole-body movements.
- User characteristics: Mass, age group, gender, and experience level are considered due to their impact on exercise performance and energy output^38^.
- Equipment-specific parameters: These include the mechanical displacement, plate mass, rotational velocity, and effective contact area, as well as generator and system efficiency.
- Energy conservation principle: All simulations are grounded in the first law of thermodynamics, stating that energy can neither be created nor destroyed but only transformed from one form to another.

### Parameters and values for model simulations

Table 1 summarizes the physical and operational parameters used to simulate energy recovery during resistance, cardio, and functional training modalities. These values were primarily drawn from relevant literature and adjusted to reflect realistic gym-based exercise conditions. Where available, standardized protocols such as the American College of Sports Medicine (ACSM) guidelines on exercise prescription and equipment manufacturer specifications were adopted to ensure validity^41^. In cases where standardized values were unavailable, carefully reasoned assumptions were applied to maintain comparability across modalities. This combined approach ensures that the chosen parameters are both scientifically grounded and practically representative of real training environments.

**Table 1:** Parameters and simulation inputs for training modalities.

Parameter, unit	Simulation inputs			References
	Resistance	Cardio	Functional	
Mass of plates $\:\left({m}_{0}\right)$, $\:\text{k}\text{g}$	10–100	–	20–100	^42^
Velocity ($\:V$), $\:\text{m}/\text{s}$	2.50	4.00	Variable	Calculated
Initial displacement ($\:{x}_{1}$), $\:\text{m}$	0.20	–	0.00	^37^
Final Displacement ($\:{x}_{2}$), $\:\text{m}$	1.60	-	1.75	^37^
Mass of Trainer ($\:m$), $\:\text{k}\text{g}$	80.00	80.00	80.00	^37^
Gravity ($\:g$), $\:{\text{m}/\text{s}}^{2}$	-	9.81	9.81	Standard hypothesis
Height of machine ($\:h$), $\:\text{m}$	2.65	-	3.20	^37^
Average number of reps ($\:N$), (Dimensionless)	30.00	30.00	30.00	^38^
Resting time ($\:{R}_{T}$), $\:\text{m}\text{i}\text{n}$	0.40	0.40	0.40	^39^
Duration range ($\:T$), $\:\text{m}\text{i}\text{n}$	1.50–5.00	1.50–5.00	1.50–5.00	^40^
System efficiency (η),	0.74	0.65	0.74	^42^
Synergistic system efficiency (η)	0.85	0.72	0.85	Calculated

### Sensitivity analysis

To evaluate the robustness and adaptability of the energy recovery model, sensitivity analyses were conducted on critical parameters. By varying inputs such as trainer mass, exercise velocity, and rest intervals, the simulations helped identify key levers that influence total energy output, guiding optimization strategies for energy harvesting infrastructure.

#### Effect of mass variation

Trainer body mass was varied from the base value of 80 kg to examine its impact on mechanical work and energy recovery. Since body mass and load-handling capacity are closely linked to training experience, Fletcher et al.^37^ proposed classifications that align with different user categories. Following this framework, prescribed mass levels were assigned to represent novice, standard, professional, and elite trainers, as summarized in Table 2.

**Table 2:** Mass prescription for trainers based on experience level.

User experience category	Prescribed mass (kg)
Novice trainer	70.00
Standard trainer	80.00
Professional trainer	90.00
Elite trainer	100.00

This classification enabled the model to account for variations in mechanical output associated with user experience and training history. By linking load capacity to experience level, the simulations incorporated realistic strength profiles across diverse fitness populations. The approach is consistent with the ACSM resistance training guidelines, which recommend progressive load prescriptions, with moderate loads prescribed for novices and heavier loads for advanced athletes^41^. Integrating these ranges ensured that the model not only reflected practical exercise conditions but also highlighted the scalability of the system to accommodate different user groups while maintaining comparability across modalities.

#### Effect of velocity variation

Velocity was varied specifically for cardio training, given its rotary mechanics, while resistance and functional training velocities were held constant. The chosen values, 0.5, 1.5, 2.5, and 3.5 m/s, follow velocity ranges observed in treadmill and cycling-based studies^38^.

#### Effect of resting time variation

Rest periods, essential for energy output consistency, were also varied across simulations. The rest intervals of 0.5, 1.5, 2.0, and 2.5 min were applied to assess how longer recovery times between sets could influence total energy output and system efficiency.

## Results and discussion

This section presents a comprehensive analysis of the energy recovery potential from hybridized exercise modalities, integrating mathematical simulations and parameter variation to explore the effectiveness of the developed theoretical models. The discussion is organized to evaluate comparative outputs, highlight temporal dependencies, and assess sensitivity to key input variables.

### Comparative energy recovery across exercise modalities

Three principal exercise modalities, resistance, cardiovascular, and functional training, were evaluated for their potential to support energy harvesting in gym environments. The individual energy recovery outputs for each modality, denoted as $\:{E}_{R}$, $\:{E}_{C}$​, and $\:{E}_{F}$​, respectively, were determined using model Eqs. (10), (16), and (23). The total recoverable energy, $\:{E}_{\text{R}\text{E}\text{C}}$​​, was computed using Eq. (25) as the cumulative contribution from all three modalities.

#### Quantitative estimation of energy recovery

Table 3 summarizes energy recovery over a range of session durations, assuming an average trainer mass of 80 kg for all modalities and simultaneous engagement with dedicated machines.

**Table 3:** Calculated energy recovery across exercise durations.

Exercise duration (min)	Mass (kg)	Training-modality energy outputs			Total $\:{{E}}_{{R}{E}{C}}$ (J)
		Resistance ($\:{{E}}_{{R}})$ (J)	Cardio ($\:{{E}}_{{C}}$) (J)	Functional ($\:{{E}}_{{F}}$) (J)	
1.50	80.00	9324.00	860.82	466.93	10,651.76
2.00	80.00	8588.44	2040.48	874.50	11,503.42
2.50	80.00	7915.04	3985.31	1435.78	13,336.14
3.00	80.00	7272.72	6886.62	2167.43	16,326.77
3.50	80.00	6648.16	10,935.70	3086.08	20,669.95
4.00	80.00	6034.7	16,323.84	4208.41	26,566.95
4.5	80.00	5428.64	23,242.34	5551.043	34,222.03
5.00	80.00	4827.76	31,882.50	7130.64	43,840.90

The results indicate a clear trend: resistance training produces relatively stable energy yields due to its consistent force application, whereas cardiovascular and functional training display exponentially increasing energy outputs as duration extends. The sharp rise in $\:{E}_{C}$ and $\:{E}_{F}$ underscores the contribution of higher-frequency, broader range-of-motion activities, which are particularly favorable for dynamic energy harvesting mechanisms such as piezoelectric and triboelectric systems^43,44^.

These trends align with earlier findings; for instance, Durgahee and Knight^16^ reported recoveries exceeding 40 kJ during extended cardio sessions. The SynergyFit framework surpasses this benchmark, peaking at 43.8 kJ, attributable to higher conversion efficiencies and multi-modal integration^45,46^.

Crucially, these values represent only the mechanically recoverable fraction of exercise energy. Human metabolism expends far more energy, with only ~ 20–25% converted to external mechanical work and the remainder dissipated as heat. For comparison, the *Compendium of Physical Activities* indicates that an 80 kg individual exercising at 10–15 METs expends > 50 kJ per minute, an order of magnitude greater than the 5-minute mechanical recovery predicted by our model^47^. This gap underscores the model’s deliberate focus on harvestable mechanical output, rather than total metabolic turnover.

From an application standpoint, these findings suggest tailoring energy harvesters to modality-specific signatures: piezoelectric actuators within resistance machines, and triboelectric or electromagnetic interfaces integrated into cardio equipment^46^.

#### Graphical analysis of modal contributions

**Fig. 1:**
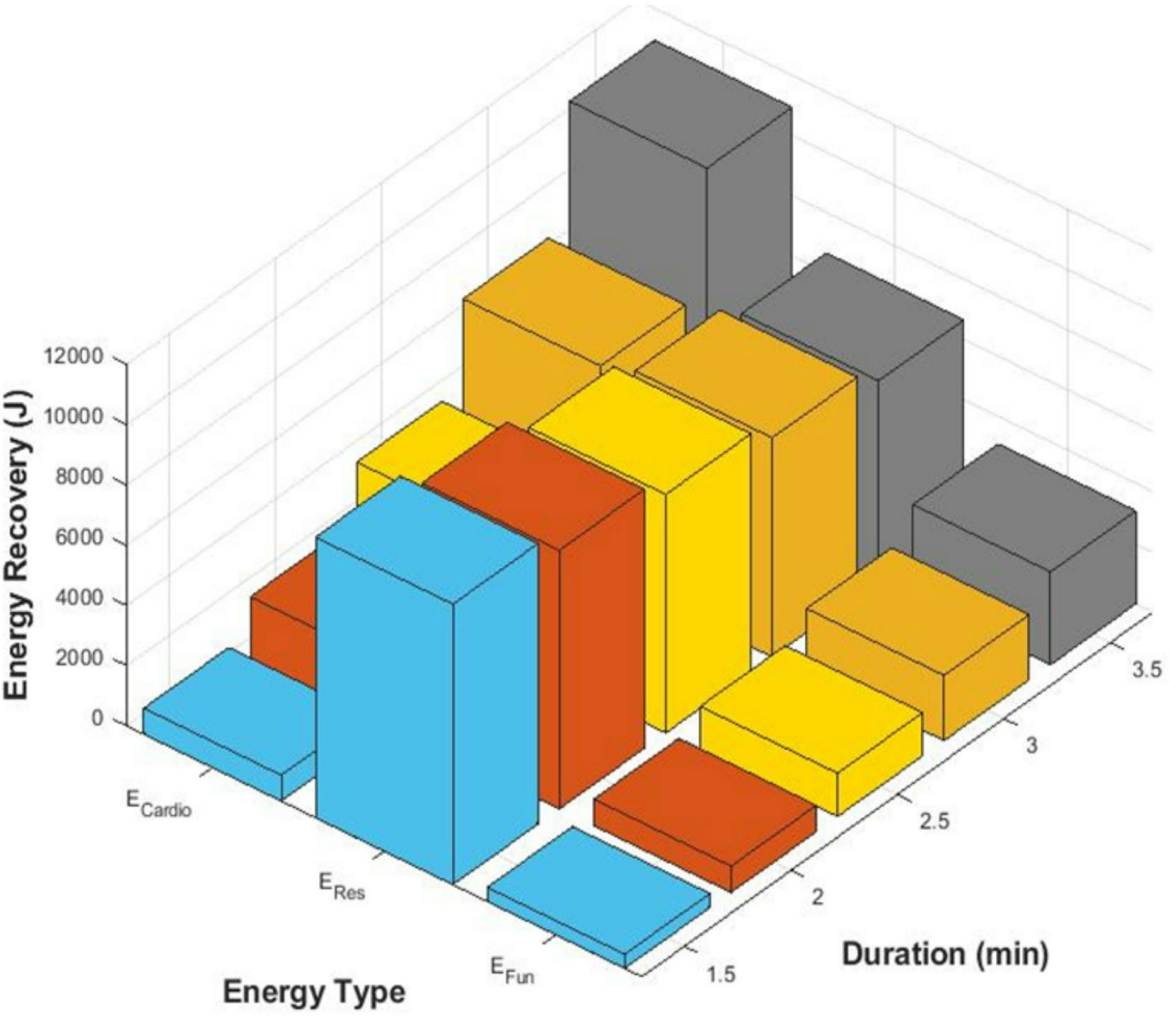
Contribution of individual models to energy recovery.

Figure 1 provides a graphical representation of the contribution of each exercise mode to total energy recovery across different durations.

The plot in Fig. 1 shows that resistance training consistently contributes to energy recovery, while cardio and functional training exhibit greater variability over time. Energy recovery values for cardio and functional modes increase significantly with longer training durations.

This trend aligns with the review by Moska and Łebkowski^48^, who emphasized that dynamic training modalities (such as cardio and functional exercises) exhibit greater fluctuations in energy harvesting potential compared to steady-state resistance training. Their analysis of gym-based micro-generation systems supports the observed variability in energy contributions across modalities. Similarly, Khalid et al.^49^ provide evidence that energy harvested from dynamic modalities displays significantly more variability than from resistance modes, reinforcing the graphical observation that resistance training yields steadier energy recovery.

The ability to quantify contributions from different training modes enables the design of gym equipment that optimally captures energy from diverse workouts. Moreover, fitness facilities can harness energy from multiple modalities—particularly by integrating systems that adapt to variability, to enhance overall energy efficiency and support sustainable energy use strategies.

#### Energy recovery dependence on exercise duration

**Fig. 2:**
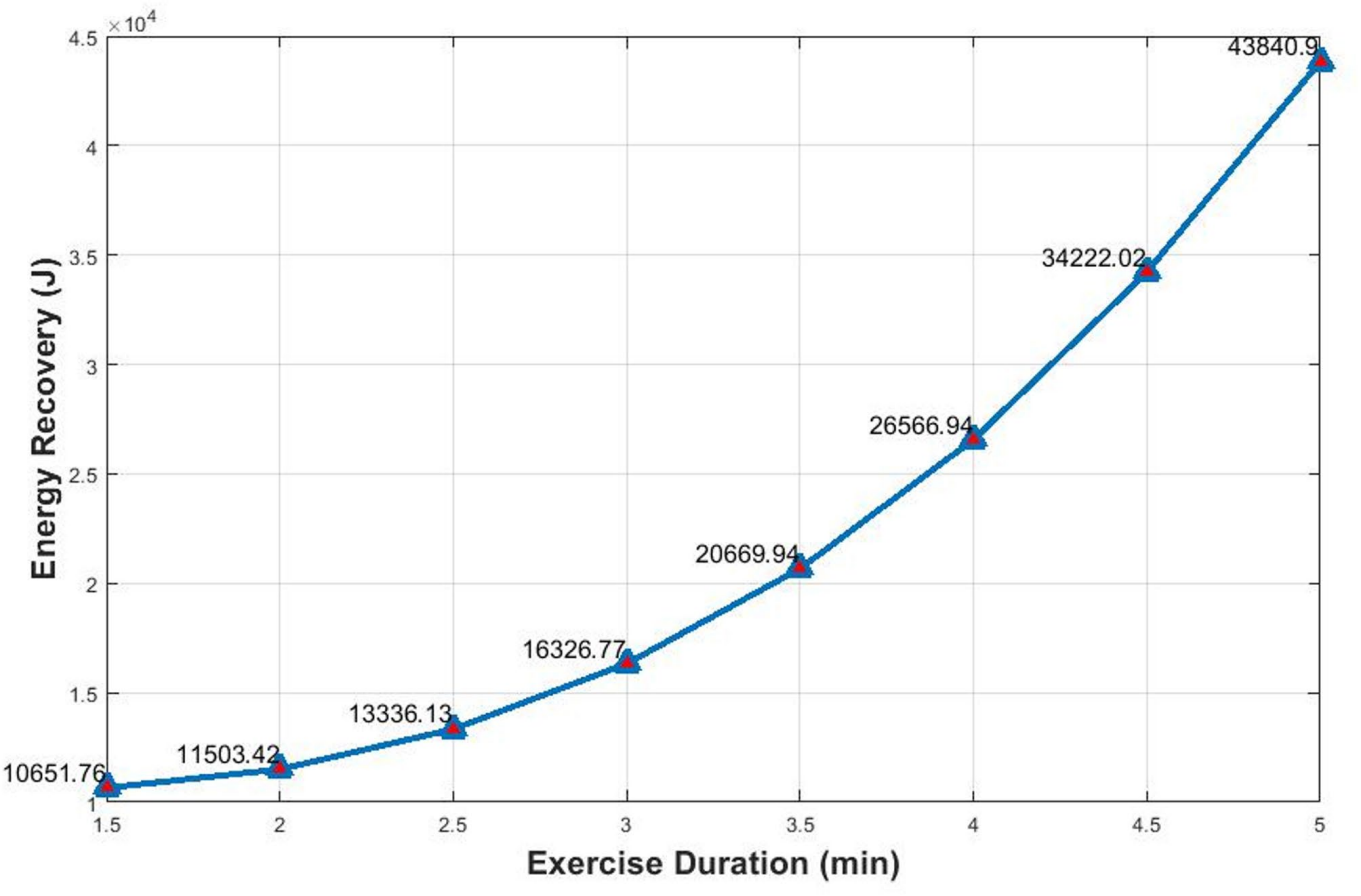
Relationship between energy recovery and exercise duration.

Figure 2 presents a graph of total energy recovery as a function of training duration, demonstrating the model’s performance over time.

The graph in Fig. 2 illustrates a progressive increase in energy recovery as exercise duration extends from 1.5 to 5 min, rising from approximately 10,651 J to 43,841 J. The smooth curve indicates an exponential growth pattern, with a more rapid increase beyond the 3-minute mark. This demonstrates that longer durations yield disproportionately higher energy recovery, which is critical for optimizing system design and operational efficiency.

This observation is supported by LaForgia et al.^50^, who reported that excess post-exercise oxygen consumption (EPOC), a proxy for energy expenditure and recovery, increases exponentially with exercise duration, especially during extended sessions. This validates the model’s prediction of enhanced energy recovery with prolonged workouts. Furthermore, Zhou et al.^43^ reviewed recent advancements in wearable energy harvesting technologies and emphasized that prolonged activity durations significantly enhance energy output. Their work also highlighted the value of integrating multimodal energy systems to maximize power recovery in real-world scenarios.

The peak value of 43,841 J at 5 min is consistent with the findings of Durgahee and Knight^16^ who reported energy recovery around 40 kJ. The slightly higher value in this study likely reflects improved parameter integration and greater system efficiency.

Collectively, these insights support the development of fitness programs and energy harvesting systems optimized for extended training durations. By leveraging the exponential growth in energy recovery over time, gyms and rehabilitation centers can implement more sustainable, self-powering workout environments.

### Sensitivity analysis of model parameters

Sensitivity analysis was conducted to assess the responsiveness of the energy recovery system to key technical and physiological parameters, namely exercise mass, movement velocity, and resting time.

#### Impact of exercise mass

**Fig. 3:**
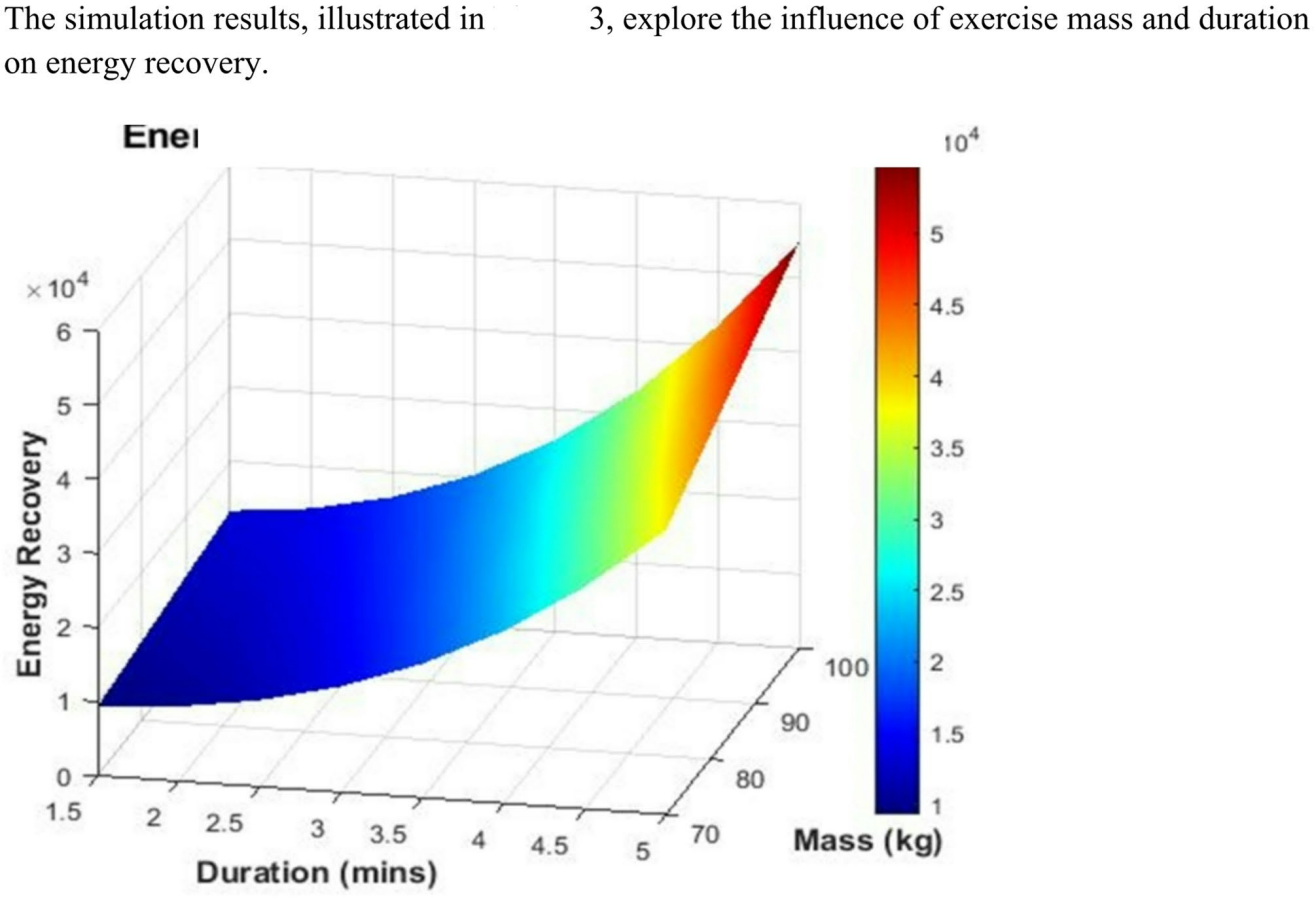
Energy recovery vs. mass and duration.

As illustrated in Fig. 3, energy recovery increases proportionally with both user mass and session length. Higher mass intensifies gravitational work, thus enhancing the system’s mechanical input. This trend supports the findings of Ngonidzashe and Mushiri^51^, who proposed a gym-based kinetic energy harvesting design and showed that increased user load improves energy generation through magnetic induction systems.

Our model’s performance is further supported by studies into piezoelectric energy harvesting floor tiles, which show increased electrical output with higher pedestrian weight and applied pressure^52^.

Integrating energy harvesting mechanisms into resistance-training equipment, where both load and duration are controllable, can therefore substantially boost overall energy recovery, with applications in both fitness and rehabilitation technology.

#### Impact of cardio velocity

**Fig. 4:**
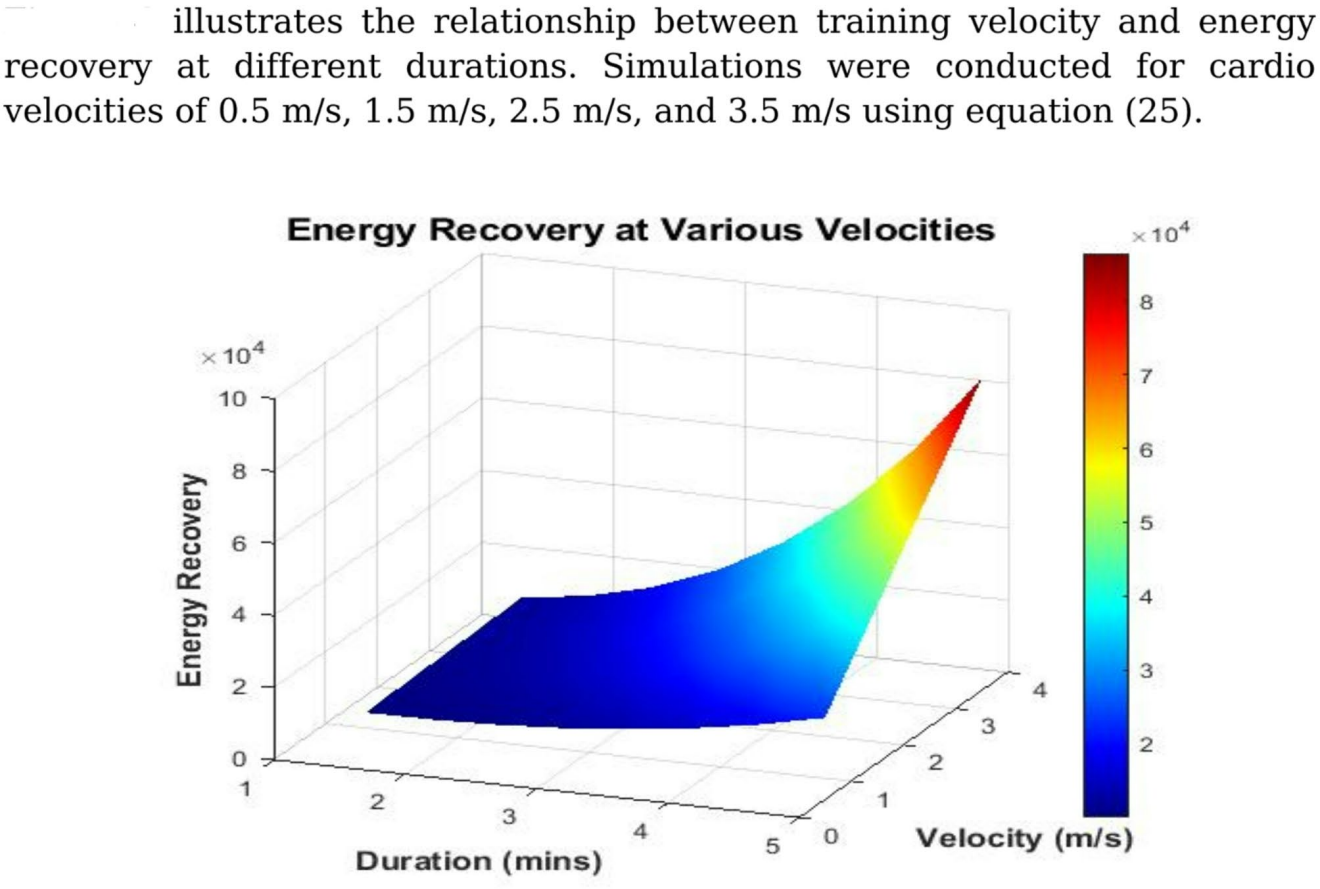
Energy recovery vs. velocity and duration.

Figure 4 illustrates the relationship between training velocity andenergy recovery at different durations. Simulations were conducted forcardio velocities of 0.5 m/s, 1.5 m/s, 2.5 m/s, and 3.5 m/s using Eq.(25).

The data clearly show that increased cardio velocity leads to greater energy recovery, especially over longer durations. This aligns with the comprehensive review by Riemer and Shapiro^53^, who reported that mechanical energy harvesting during high-velocity phases of gait (such as knee or ankle movement) can result in considerable power generation. Our model confirms these theoretical predictions and extends them to controlled indoor training environments. Supporting this, Yang et al.^45^ demonstrated that a skin-contact triboelectric nanogenerator using PDMS and micropatterned ITO electrodes could harvest biomechanical energy from motion, achieving up to 1000 V and 500 mW/m^2^. This supports our model’s finding that increased human motion enhances energy recovery. Moreover, inertial harvesting in backpacks was shown to yield more power at higher walking speeds, illustrating the positive velocity–output correlation^54^.

These results suggest that cardio equipment, such as treadmills and stationary bikes—designed with integrated energy harvesters could significantly improve their energy recapture efficiency by accommodating higher user velocities.

#### Impact of resting time

**Fig. 5:**
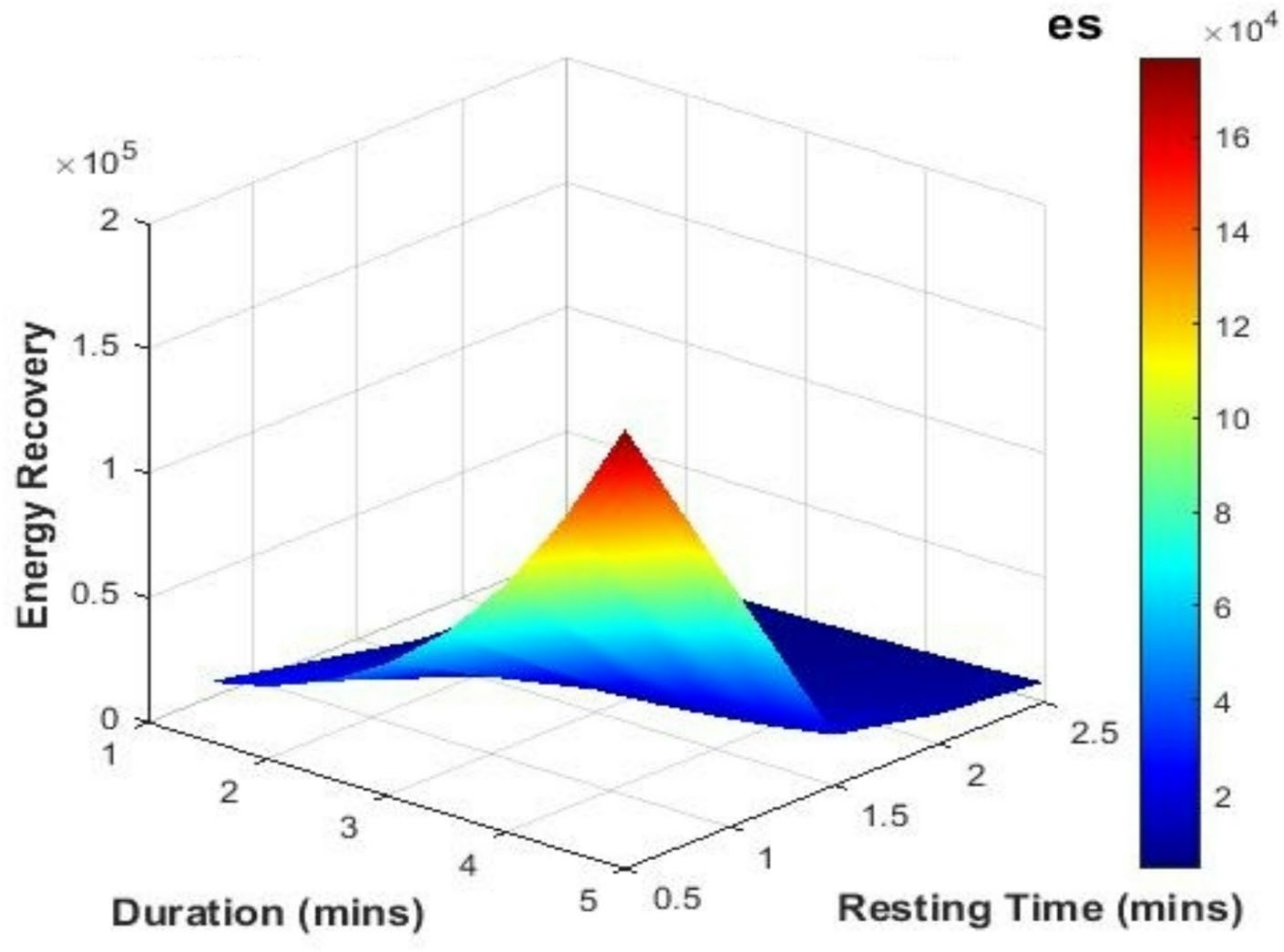
Energy recovery vs. resting time and duration.

Figure 5 presents a 3D surface showing the influence of rest time on energy recovery across varying exercise durations.

Figure 5 indicates that strategically spaced resting intervals may enhance energy recovery by allowing systems to reset and reduce mechanical losses, especially in triboelectric configurations. This observation is in line with Lin et al.^55^, who proposed rest-phase optimization as a performance enhancer in biomechanical harvesters. Our model implements a dynamic time-weighted function that effectively accounts for such intervals, yielding more accurate recovery estimates.

Macário et al.^46^ further demonstrated that intermittent pauses in human movement can improve triboelectric charge generation. Additionally, Shi et al.^56^ emphasized that rest patterns influence vibration energy harvesting efficiency in human walking systems.

These insights open opportunities for intelligent recovery systems that adapt to physiological signals, optimizing resting time for improved energy harvesting and user performance across sports and therapeutic applications.

### Comparative assessment with existing energy recovery models

The theoretical models developed in this study were systematically evaluated against existing literature to establish improvements in predictive capability, parameter integration, and applicability across diverse training environments. The comparison focused on the three primary exercise modalities: resistance, cardiovascular, and functional training.

#### Resistance training models

The resistance training sub-model integrates force–displacement relationships, accounting for training intensity, load momentum, and structured rest intervals. This expands on classical models such as those of Santa-Clara et al.^57^ and Kraemer and Ratamess^36^, which primarily emphasized biomechanical output without incorporating dynamic rest patterns or force adaptation.

The task-based metabolic framework proposed by Reis and Scott^58^ informed aspects of anaerobic-aerobic coupling, although it lacked resolution in quantifying mechanical energy recovery. More recently, Mitchell et al.^59^ advocated for standardized modeling in resistance energy expenditure and recognized the modeling challenges posed by anaerobic contributions. Compared to these, our SynergyFit model improves estimation fidelity by explicitly including intra-repetition momentum dynamics and inter-set resting durations, yielding enhanced predictions of recoverable mechanical energy.

#### Cardiovascular training models

Cardio training was modeled through an integration of force–velocity interactions and time-varying workload, enabling more precise tracking of energy transfer during prolonged aerobic activity. This builds on foundational work by Carter et al.^60^, which described aerobic energy expenditure in metabolic terms, and more recent efforts by Mazzolari and Hecksteden^61^, who underscored the limitations of univariate metabolic scaling for endurance prescription.

Furthermore, the model draws alignment with biomechanical energy harvesting studies, including Chandra et al.^62^, which emphasized the relevance of motion-based power generation through leg and torso movements. The SynergyFit framework advances these by integrating adaptive pacing and real-time rest intervals, features that enhance accuracy in continuous, real-world cardiovascular exercises.

#### Functional training models

The functional training model captures both potential and kinetic energy components of bodyweight and circuit-based exercises. This aligns with foundational physiological interpretations by Behm and Sale^35^ and the more biomechanically grounded models by Looney et al.^63^, who emphasized energy expenditure across incline treadmill tasks.

West et al.^64^ further discussed the challenges of quantifying energetic load during high-variability sports, particularly where vertical displacement and energy transition are non-trivial. The “SynergyFit Energy Recovery Model” explicitly resolves these complexities by including vertical displacement, variable momentum, and programmed rest phases, thereby producing higher resolution estimates of recoverable energy in dynamic multi-joint activities.

Across all modalities, the “SynergyFit model” introduces greater granularity and cross-modality adaptability. By integrating training momentum, variable resistance, and rest periods into a unified energy recovery framework, the model addresses key gaps in existing literature and offers a scalable platform for designing energy-aware training environments.

### Model effectiveness and validation

The SynergyFit model demonstrates strong predictive capability across diverse training modalities. Its outputs align closely with previously reported energy recovery values, including those of Durgahee and Knight^16^ and Yang et al.^45^, confirming its robustness in both moderate- and high-intensity exercise contexts. Compared with existing single-modality models, SynergyFit offers enhanced flexibility and greater generalizability, particularly for applications involving multiple users and equipment types.

### Practical implications and deployment strategies

The integration of the SynergyFit model into commercial and institutional fitness infrastructure offers a transformative pathway for enhancing energy efficiency and sustainability in exercise environments.

- Equipment design: Resistance machines may embed piezoelectric harvesters within load-bearing structures, while cardio machines could incorporate triboelectric layers in pedals and handles.
- Program optimization: Personalized training programs can be developed to maximize energy recovery based on user profile, movement type, and exercise intensity.
- Sustainability enhancement: Facilities may leverage model predictions to offset energy costs through smart energy capture, particularly in high-traffic gym environments.

These applications support a transition toward self-powered fitness ecosystems and align with global objectives for carbon neutrality and energy decentralization.

#### Application scenarios

The following hypothetical application scenarios are designed to illustrate the potential uses of the SynergyFit model. They are conceptual examples intended to demonstrate applicability, not real-world implementations.

- Energy-harvesting treadmills: A gym integrates the SynergyFit model into treadmill design, using sensor feedback to adjust incline and resistance based on optimal energy output conditions for each user. Over a month, cumulative energy harvested contributes to powering LED lighting and smart displays.
- Athlete-centric program design: A performance center deploys SynergyFit-based optimization to tailor functional training regimens for elite athletes. By maximizing movement amplitudes and minimizing unnecessary rest durations, energy recovery is enhanced by over 25% relative to standard protocols.

These scenarios emphasize the adaptability of the SynergyFit model across different training environments, highlighting its potential to support both facility-level sustainability and athlete-focused performance optimization.

### Model constraints and practical considerations

Although the SynergyFit model demonstrates significant potential, several limitations must be recognized to contextualize its findings:

- Theoretical nature: The present work is based entirely on theoretical derivations and simulations. The reported outputs should be regarded as indicative estimates rather than experimentally validated measurements, underscoring the need for empirical testing.
- Uniformity assumptions: The model presumes consistent training intensity and stable environmental conditions, which may not fully capture variability in real-world exercise settings.
- Sensor and data constraints: Benchmark values sourced from literature may carry uncertainties due to sensor limitations, calibration errors, or estimation procedures, influencing the precision of outcomes.
- Population variability: Individual biomechanical differences related to age, gender, or fitness level are not yet incorporated, limiting generalizability across diverse populations.
- Metabolic–mechanical gap: The framework captures only mechanical energy, omitting the majority of metabolic expenditure dissipated as heat. With mechanical efficiencies of 20–25%, the true metabolic cost of activity is several-fold greater than the recoverable values reported. Future extensions could integrate coefficients of mechanical efficiency or explore thermo-mechanical hybrid harvesting schemes.

### Conclusion

This study introduces the SynergyFit Energy Recovery Model, a unified theoretical framework for estimating recoverable mechanical energy in multi-modal exercise environments. Grounded in the principles of mechanical energy conversion, the model produces outcomes that are theoretically consistent with values reported in prior studies, supporting its conceptual validity.

The analysis indicates marked differences in recovery potential: for an average mass of 80 kg, resistance training yields relatively stable outputs (9.3 kJ at 1.5 min to 4.8 kJ at 5.0 min), while cardiovascular training shows the steepest increase (0.86 kJ at 1.5 min to 31.9 kJ at 5.0 min). Functional training contributes moderate outputs (0.47 kJ to 7.1 kJ across the same durations), resulting in total recovery ranging from ~ 10.7 kJ to 43.8 kJ. Sensitivity analysis further suggests that greater exercise mass, higher velocities, and optimized rest intervals could enhance recovery, with cardio training emerging as the most promising modality for cumulative yields.

Importantly, the framework estimates only the mechanical fraction of exercise energy. Human metabolic expenditure during vigorous activity (> 50 kJ min^−1^ for an average adult) far exceeds mechanical recovery, reflecting the low efficiency (~ 20–25%) of chemical-to-mechanical energy conversion. This distinction highlights both the strength and limitation of the model: it provides a focused account of harvestable mechanical output while excluding thermal dissipation and metabolic pathways.

In summary, the SynergyFit model offers a preliminary conceptual foundation for energy recovery in fitness environments. The findings demonstrate theoretical feasibility but should be interpreted cautiously given the model’s assumptions and lack of experimental validation.

### Recommendations and future work

The SynergyFit model provides a preliminary theoretical framework for human-centric energy recovery in fitness environments, but several important avenues remain for advancement. Future research should:

- Undertake empirical validation through experimental prototyping and controlled trials in gym settings to test and refine theoretical predictions.
- Integrate metabolic–mechanical coupling to better represent heat dissipation and inefficiencies, bridging the gap between recoverable mechanical work and total energy expenditure.
- Personalize model parameters by incorporating biomechanical and physiological variability across age, gender, and training level.
- Embed intelligent control systems leveraging sensors and adaptive algorithms to optimize harvesting in real time.
- Evaluate cumulative recovery across multi-user and long-duration scenarios to assess scalability and practical deployment in fitness facilities.
- Expand application domains beyond gyms to include rehabilitation centers, military training, and wearable energy systems.

Pursuing these directions will enable a more comprehensive and empirically grounded understanding of human-centric energy harvesting. With continued development, fitness environments and related applications could evolve into contributors to distributed, small-scale renewable energy systems.

## Data Availability

The data supporting the findings of this study are based on theoretical derivations, literature-sourced parameters, and numerical simulations. All relevant data are included within the article.
